# Exercise-based cardiac rehabilitation after stroke and the risk of incident dementia: an observational study from a global federated network

**DOI:** 10.1093/esj/aakag127

**Published:** 2026-10-22

**Authors:** Leandra Serio, Amir Askarinejad, Chen Wang, Koohyar Ahmadzadeh, Tianshu Gu, Riccardo Proietti, Claudio Ferri, Ben Buckley, Gregory Y H Lip

**Affiliations:** Institute of Life Course and Medical Sciences, University of Liverpool, Liverpool, United Kingdom; Department of Life, Health & Environmental Sciences, University of L'Aquila, L'Aquila, Italy; Internal Medicine and Nephrology Division, ASL1 Avezzano-Sulmona-L'Aquila, San Salvatore Hospital, L'Aquila, Italy; Institute of Life Course and Medical Sciences, University of Liverpool, Liverpool, United Kingdom; Institute of Life Course and Medical Sciences, University of Liverpool, Liverpool, United Kingdom; Institute of Life Course and Medical Sciences, University of Liverpool, Liverpool, United Kingdom; Institute of Life Course and Medical Sciences, University of Liverpool, Liverpool, United Kingdom; Tianjin Key Laboratory of Ionic-Molecular Function of Cardiovascular Disease, Department of Cardiology, Tianjin Institute of Cardiology, The Second Hospital of Tianjin Medical University, Tianjin 300211, China; Institute of Life Course and Medical Sciences, University of Liverpool, Liverpool, United Kingdom; Department of Life, Health & Environmental Sciences, University of L'Aquila, L'Aquila, Italy; Internal Medicine and Nephrology Division, ASL1 Avezzano-Sulmona-L'Aquila, San Salvatore Hospital, L'Aquila, Italy; Institute of Life Course and Medical Sciences, University of Liverpool, Liverpool, United Kingdom; Cardiovascular Health Sciences, Research Institute for Sport and Exercise Sciences, Liverpool John Moores University, Liverpool, United Kingdom; Institute of Life Course and Medical Sciences, University of Liverpool, Liverpool, United Kingdom; Department of Clinical Medicine, Aalborg University, Aalborg, Denmark; Department of Clinical Medicine, Medical University of Bialystok, Bialystok, Poland

**Keywords:** cardiovascular risk, dementia, cognitive decline, exercise-based cardiac rehabilitation, stroke

## Abstract

**Introduction:**

Dementia is a common and disabling complication after stroke. Exercise-based cardiac rehabilitation (EXCR) may influence vascular and inflammatory mechanisms relevant to cognitive decline. We investigated the association between EXCR initiation after stroke and incident dementia.

**Patients and methods:**

Using TriNetX, we identified adults with ischaemic stroke or TIA. Patients with recorded ICD-10-CM codes indicating an NIHSS score ≥ 16 were excluded from the primary analysis. Patients initiating EXCR within 6 months were compared with those without documented post-index EXCR. Propensity score matching balanced demographics, comorbidities, medication use and available stroke-severity measures. Outcomes were assessed at 3 and 5 years.

**Results:**

The unmatched cohorts included 4226 patients initiating EXCR (mean age, 64.7 years; 60.8% male) and 1,889,796 without EXCR (mean age, 66.3 years; 48.2% male). After matching, 4138 patients remained in each cohort. At 5 years, EXCR was associated with lower hazards of incident dementia (HR, 0.70; 95% CI, 0.59–0.82), all-cause mortality (HR, 0.50; 95% CI, 0.45–0.55) and recurrent stroke (HR, 0.75; 95% CI, 0.61–0.91), but a higher hazard of rehospitalisation (HR, 1.21; 95% CI, 1.06–1.38); no significant association was observed for acute myocardial infarction. The association with incident dementia remained consistent across sensitivity analyses.

**Discussion:**

The observed association with lower dementia risk suggests that the potential benefits of post-stroke EXCR may extend beyond cardiovascular and functional outcomes.

**Conclusions:**

EXCR initiation after ischaemic stroke or TIA was associated with a lower hazard of incident dementia. This association remained consistent across the 3-year, 30-day landmark, 1-year lagged and all-stroke-severity sensitivity analyses.

## Graphical abstract

**Graphical abstract:**
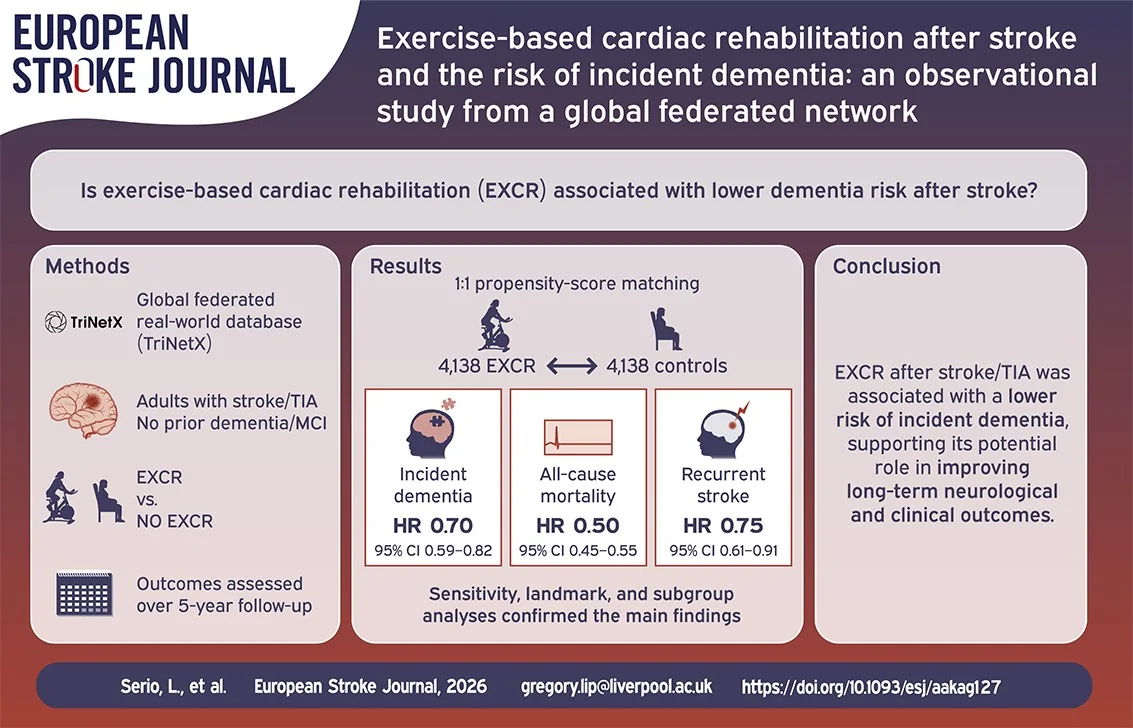

## Introduction

Stroke is a leading cause of disability and mortality worldwide and is increasingly recognised as a major risk factor for cognitive decline and dementia.^1^ Stroke survivors have a substantially increased risk of developing dementia, particularly during the early post-stroke period, although the excess risk remains elevated over time, with a significant impact on quality of life, functional independence and healthcare costs.^2^^,^^3^ Cognitive impairment after stroke has also been associated with long-term functional dependence and less favourable recovery patterns.^4^^,^^5^ Despite this, effective strategies to prevent post-stroke cognitive decline remain limited and are not yet well established, particularly given the heterogeneous clinical presentation and prognosis of post-stroke cognitive impairment,^6^ which requires a multidisciplinary approach.

Exercise-based cardiac rehabilitation (EXCR) is a well-established multidisciplinary intervention that combines structured exercise training, cardiovascular risk factor management and lifestyle counselling, and is recommended in international guidelines for cardiovascular disease.^7^^,^^8^ Contemporary rehabilitation frameworks emphasise that post-stroke recovery is a dynamic process supported by neuroplasticity and targeted rehabilitation interventions, with structured physical training representing a principal component of functional recovery.^9^ In this context, structured and progressive rehabilitation programs may contribute to favourable clinical outcomes after stroke. Beyond improvements in physical function, emerging evidence suggests that exercise-based interventions may influence cognitive outcomes through cardiovascular–brain interactions and other biological pathways involved in neuroplasticity and cognitive resilience. However, evidence regarding the effects of EXCR on long-term clinical outcomes after stroke remains limited, particularly with respect to incident dementia. Interventional studies have suggested that exercise programs may improve cognitive function in patients with stroke.^10^ Given that post-stroke recovery follows distinct temporal trajectories, we focused on EXCR initiated within 6 months after the index cerebrovascular event; this prespecified window should not be interpreted as acute or very early rehabilitation.^11^^,^^12^ Accordingly, the aim of the present study was to evaluate, in a large real-world observational cohort, the association between initiation of EXCR within 6 months after stroke and the risk of incident dementia and other clinically relevant outcomes among patients without recorded ICD-10-CM codes indicating NIHSS scores ≥ 16. An additional sensitivity analysis included all eligible patients without applying an NIHSS-based exclusion.^13^

## Patients and methods

### Study design and data source

A retrospective observational cohort study was conducted using the TriNetX Global Collaborative Network, a federated real-world research platform containing de-identified patient-level electronic health record data from participating healthcare organisations. The network includes healthcare organisations across multiple geographic regions, including the Americas, Europe, the Middle East, Africa and the Asia–Pacific region, thereby providing a geographically diverse study population. Diagnoses and treatments were identified using International Classification of Diseases, Tenth Revision (ICD-10), RxNorm, VA Drug Classification and Current Procedural Terminology (CPT) codes.

### Ethics approval and consent to participate

The study was conducted using the TriNetX LIVE research network,^14^ which provides only de-identified patient-level data in compliance with the HIPAA Privacy Rule (45 CFR § 164.514[b][1]). Data are available exclusively as aggregated counts and statistical summaries, with no personal identifiers. The de-identification process has been certified by a qualified expert (last updated December 2020); therefore, analyses on TriNetX are not considered human subjects research and are exempt from Institutional Review Board (IRB) oversight. No identifiable data were accessed or re-identified. Accordingly, this study was exempt from IRB review and informed consent requirements. All analyses complied with applicable regulations and the Declaration of Helsinki.

### Study population

Searches on TriNetX were conducted on 4 September 2026. Adult patients (≥18 years) with a diagnosis of ischaemic stroke or TIA between 1 January 2000 and 31 December 2020, were identified. Cerebrovascular events were defined using ICD-10-CM codes I63 (cerebral infarction) and G45 (transient cerebral ischaemic attacks and related syndromes). For the primary analysis, patients with recorded ICD-10-CM codes indicating NIHSS scores ≥16 were excluded (Table S1). This cutoff was informed by a published NIHSS classification in which scores of 11–15 were categorised as moderate and scores of 16–20 as severe, while recognising that alternative NIHSS categorisations have used different thresholds.^15^^,^^16^ Patients without a recorded NIHSS code were not excluded on the basis of stroke severity.

Patients were divided into 2 cohorts: (1) cohort I (EXCR group), comprising patients with a recorded EXCR-related service between 1 day and 6 months after stroke or TIA; and (2) cohort II (non-EXCR group), comprising patients with no recorded exposure to the prespecified EXCR codes at any time after the index cerebrovascular event. Exercise-based cardiac rehabilitation exposure was defined using CPT and HCPCS procedure codes and ICD-10-CM exercise counselling codes (Table S2). The absence of these exposure codes did not necessarily indicate the absence of other forms of neurological or multidisciplinary post-stroke rehabilitation.

### Index event

The index event was defined as the first recorded ischaemic stroke or TIA meeting the study inclusion criteria between 1 January 2000 and 31 December 2020.

### Outcomes

The primary outcome was incident dementia, defined by the presence of any of the following ICD-10-CM codes: Alzheimer disease (G30), vascular dementia (F01) or unspecified dementia (F03). Secondary outcomes included all-cause mortality, acute myocardial infarction (I21), stroke recurrence (I63), rehospitalisation (CPT 1013659), Alzheimer disease (G30), vascular dementia (F01) and unspecified dementia (F03). For incident outcomes, patients with a prior history of the outcome before the index date (or before the start of the time window) were excluded, ensuring that only new-onset events were captured during follow-up.

### Follow-up timing

Primary analyses were conducted over 1 day to 3 years and 1 day to 5 years. To address potential biases related to early post-stroke events and reverse causation, additional sensitivity analyses incorporating a blanking period were performed (1 month to 3 years and 1 month to 5 years). A lagged analysis was conducted by restricting follow-up from 1 year to 5 years, to further reduce potential reverse causation and early event bias. Sensitivity analyses were restricted to this lagged time window to ensure a more stable risk period and minimise confounding from early post-event dynamics.

### Statistical analysis

The causal contrast of interest was the effect of initiating EXCR within 6 months after the index cerebrovascular event, compared with not initiating EXCR, on the subsequent risk of incident dementia among patients meeting the study eligibility criteria. Interpretation of the estimated associations as causal would require consistency, positivity, conditional exchangeability given the measured baseline covariates, accurate classification and temporal ordering of exposure and outcomes and non-informative censoring.

Baseline characteristics were compared between patients who initiated EXCR and those who did not. To reduce measured baseline confounding, propensity scores were estimated using logistic regression. Covariates were selected a priori based on clinical knowledge, published evidence regarding factors associated with post-stroke dementia and participation in rehabilitation and their availability and coding reliability within the TriNetX platform. The propensity score model included demographic characteristics, cardiovascular and metabolic comorbidities, other relevant clinical conditions, medication use and available ICD-10-CM codes corresponding to NIHSS categories as a proxy for baseline stroke severity. The revised model additionally incorporated heart failure, neoplasms and personal history of nicotine dependence as a proxy for smoking history to address further potential sources of baseline confounding. Only covariates recorded before the index date were included, whereas post-index variables that could potentially represent mediators of the association between EXCR and subsequent outcomes were not incorporated into the propensity score model. Patients were matched 1:1 using nearest-neighbour matching. Complete definitions and codes for the included covariates are provided in Table S1.

Covariate balance after matching was assessed using standardised mean differences, with values < 0.10 considered indicative of adequate balance. Time-to-event analyses were performed to evaluate the associations between EXCR and clinical outcomes. Kaplan–Meier methods were used to estimate event-free survival, and groups were compared using log-rank tests. Hazard ratios (HRs) with 95% CIs were estimated using Cox proportional hazards models.

The proportional hazards assumption was formally assessed for the primary outcome at 3 and 5 years. As a complementary fixed-time analysis, the risks of incident composite dementia were compared between the propensity score matched groups at 3 and 5 years using risk ratios and odds ratios with 95% CIs.

#### Sensitivity analyses

To assess the robustness of the findings and address potential time-related biases, several sensitivity analyses were conducted, including analyses with different follow-up durations (3-year and 5-year), a 30-day landmark analysis and a 1-year lagged analysis. To minimise potential confounding from early post-stroke events, an additional sensitivity analysis was conducted in which outcomes were evaluated 1 year after the index event through 5 years after the index event.

Finally, to assess whether the primary findings were dependent on the restriction according to baseline stroke severity, an additional sensitivity analysis included all eligible patients with ischaemic stroke or TIA, without exclusion based on NIHSS severity. For this analysis, propensity score matching was repeated using the revised covariate set, including the available ICD-10-CM-based NIHSS categories, and incident composite dementia was evaluated at 3 and 5 years.

All analyses were conducted within the TriNetX platform, which integrates R- and Python-based statistical packages. Missing data were not imputed, and analyses were performed on available data. All tests were 2-sided, with *P* < .05 considered statistically significant.

#### Subgroup analysis

Subgroup analyses were conducted according to age (≥75 vs  ≤74 years), sex, hypertension, diabetes mellitus, clinically identified heart disease, atrial fibrillation, peripheral artery disease and chronic kidney disease.

## Results

### Study population and baseline characteristics

Before propensity score matching, the final cohorts included 4226 patients in the EXCR group (mean age 64.7 ± 13.5 years; 60.8% male) and 1,889,796 patients in the non-EXCR group (mean age 66.3 ± 15.5 years; 48.2% male). Before propensity score matching (PSM), a higher prevalence of comorbidities was observed in the EXCR group, including ischaemic heart disease (71.6% vs 16.4%), hypertension (87.8% vs 37.9%), dyslipidaemia (78.0% vs 27.4%) and atrial fibrillation (36.1% vs 8.9%) (Table 1).

**Table 1:** Baseline characteristics before and after propensity score matching in the primary analysis at 5 years.

	Before PSM				After PSM			
Characteristic	EXCR (*n* = 4,226)	Non-EXCR (*n* = 1,889,796)	*P* value	Std diff	EXCR (*n* = 4,138)	Non-EXCR (*n* = 4138)	*P* value	Std diff
**Demographics**								
**Age at index, years**	64.7 ± 13.5	66.3 ± 15.5	<.001	0.107	64.8 ± 13.5	65.2 ± 14.2	.186	0.029
**White**	3,350 (79.6%)	1,046,690 (58.9%)	<.001	0.460	3,287 (79.4%)	3,294 (79.6%)	.849	0.004
**Male**	2,558 (60.8%)	856,817 (48.2%)	<.001	0.254	2,504 (60.5%)	2,509 (60.6%)	.910	0.002
**Diagnoses**								
**Hypertensive diseases**	3,694 (87.8%)	673,860 (37.9%)	<.001	1.204	3,626 (87.6%)	3,699 (89.4%)	.012	0.055
**Dyslipidaemia**	3,281 (78.0%)	486,952 (27.4%)	<.001	1.174	3,218 (77.8%)	3,285 (79.4%)	.073	0.039
**Ischaemic heart diseases**	3,015 (71.6%)	290,443 (16.4%)	<.001	1.341	2,958 (71.5%)	3,064 (74.0%)	.009	0.058
**Diabetes mellitus**	1,785 (42.4%)	315,956 (17.8%)	<.001	0.557	1,751 (42.3%)	1,798 (43.5%)	.297	0.023
**Atrial fibrillation and flutter**	1,521 (36.1%)	157,825 (8.9%)	<.001	0.690	1,481 (35.8%)	1,451 (35.1%)	.491	0.015
**Chronic kidney disease**	1,039 (24.7%)	153,976 (8.7%)	<.001	0.440	1,022 (24.7%)	1,107 (26.8%)	.033	0.047
**Other peripheral vascular diseases**	566 (13.5%)	82,270 (4.6%)	<.001	0.311	561 (13.6%)	576 (13.9%)	.632	0.011
**Heart failure**	1,726 (41.0%)	146,913 (8.3%)	<.001	0.821	1,686 (40.7%)	1,741 (42.1%)	.220	0.027
**Neoplasms**	1,364 (32.4%)	344,186 (19.4%)	<.001	0.301	1,349 (32.6%)	1,368 (33.1%)	.656	0.010
**Personal history of nicotine dependence**	1,499 (35.6%)	115,822 (6.5%)	<.001	0.764	1,465 (35.4%)	1,443 (34.9%)	.612	0.011
**Mild cognitive impairment**	108 (2.6%)	14,635 (0.8%)	<.001	0.135	108 (2.6%)	94 (2.3%)	.319	0.022
**NIHSS score**								
**NIHSS score 0–9**	772 (18.3%)	2,174 (0.1%)	<.001	0.663	707 (17.1%)	681 (16.5%)	.444	0.017
**NIHSS score 10**	21 (0.5%)	89 (0.0%)	<.001	0.099	18 (0.4%)	28 (0.7%)	.139	0.033
**NIHSS score 11**	28 (0.7%)	70 (0.0%)	<.001	0.115	21 (0.5%)	17 (0.4%)	.515	0.014
**NIHSS score 12**	14 (0.3%)	59 (0.0%)	<.001	0.080	14 (0.3%)	12 (0.3%)	.694	0.009
**NIHSS score 13**	12 (0.3%)	60 (0.0%)	<.001	0.074	12 (0.3%)	10 (0.2%)	.669	0.009
**NIHSS score 14**	16 (0.4%)	58 (0.0%)	<.001	0.086	14 (0.3%)	12 (0.3%)	.694	0.009
**NIHSS score 15**	23 (0.5%)	54 (0.0%)	<.001	0.104	17 (0.4%)	15 (0.4%)	.723	0.008
**Medications**								
**Platelet aggregation inhibitors**	3,692 (87.7%)	398,613 (22.4%)	<.001	1.740	3,623 (87.6%)	3,635 (87.8%)	.688	0.009
**Anticoagulants**	3,685 (87.6%)	360,421 (20.3%)	<.001	1.829	3,615 (87.4%)	3,631 (87.7%)	.594	0.012
**Antilipemic agents**	3,581 (85.1%)	421,675 (23.7%)	<.001	1.564	3,512 (84.9%)	3,539 (85.5%)	.403	0.018
**Beta blockers/related**	3,397 (80.7%)	419,619 (23.6%)	<.001	1.393	3,329 (80.4%)	3,358 (81.2%)	.418	0.018
**Diuretics**	2,791 (66.3%)	352,221 (19.8%)	<.001	1.063	2,736 (66.1%)	2,829 (68.4%)	.029	0.048
**Calcium channel blockers**	2,465 (58.6%)	295,094 (16.6%)	<.001	0.961	2,407 (58.2%)	2,462 (59.5%)	.219	0.027
**ACE inhibitors**	1,985 (47.2%)	279,053 (15.7%)	<.001	0.720	1,950 (47.1%)	2,020 (48.8%)	.124	0.034
**Antihypertensives, other**	1,693 (40.2%)	194,633 (11.0%)	<.001	0.712	1,664 (40.2%)	1,744 (42.1%)	.074	0.039
**Angiotensin II inhibitors**	1,056 (25.1%)	162,133 (9.1%)	<.001	0.434	1,031 (24.9%)	1,084 (26.2%)	.182	0.029

Abbreviations: ACE=angiotensin-converting enzyme; EXCR=exercise-based cardiac rehabilitation; PSM=propensity score matching.

After PSM, 4138 patients remained in each cohort, and baseline characteristics were well balanced, with all standardised mean differences below 0.10, including age, sex, vascular comorbidities, heart failure, neoplasms, personal history of nicotine dependence, mild cognitive impairment and available NIHSS categories (Table 1).

### Survival analysis

#### Three-year outcomes

Before PSM, EXCR was associated with lower hazards of composite dementia (HR, 0.77; 95% CI, 0.67–0.90), Alzheimer’s disease (HR, 0.61; 95% CI, 0.45–0.83), unspecified dementia (HR, 0.80; 95% CI, 0.68–0.94) and all-cause mortality (HR, 0.68; 95% CI, 0.62–0.74) compared with no EXCR (Table 2; Figure 1). After PSM, the EXCR group had lower hazards of composite dementia (HR, 0.67; 95% CI, 0.55–0.82), Alzheimer’s disease (HR, 0.65; 95% CI, 0.44–0.98), vascular dementia (HR, 0.68; 95% CI, 0.50–0.91), unspecified dementia (HR, 0.63; 95% CI, 0.50–0.78), all-cause mortality (HR, 0.44; 95% CI, 0.39–0.49) and stroke recurrence (HR, 0.71; 95% CI, 0.57–0.88) compared with the non-EXCR group (Table 2; Figure 1). No significant between-group differences were observed for acute myocardial infarction (AMI) or rehospitalisation after PSM.

**Table 2:** Hazard ratios comparing EXCR group with non-EXCR group at 3 and 5 years.

	Before PSM			After PSM		
3-year outcomes	EXCR group	Non-EXCR group	HR (95% CI)	EXCR group	Non-EXCR group	HR (95% CI)
**Composite dementia**	177/4,212 (4.20%)	88,757/1,898,781 (4.67%)	0.77 (0.67–0.90)	167/4,005 (4.17%)	222/3,832 (5.79%)	0.67 (0.55–0.82)
**All-cause mortality**	439/4,348 (10.10%)	259,634/2,013,842 (12.89%)	0.68 (0.62–0.74)	420/4,138 (10.15%)	875/4,138 (21.15%)	0.44 (0.39–0.49)
**AMI**	218/2,851 (7.65%)	59,926/1,900,472 (3.15%)	2.14 (1.87–2.44)	208/2,737 (7.60%)	228/3,012 (7.57%)	0.95 (0.79–1.15)
**Stroke recurrence**	136/1,041 (13.06%)	62,321/551,382 (11.30%)	1.07 (0.91–1.27)	128/990 (12.93%)	215/1,243 (17.30%)	0.71 (0.57–0.88)
**Rehospitalisation**	298/1,206 (24.71%)	232,430/1,682,408 (13.82%)	1.64 (1.47–1.84)	292/1,184 (24.66%)	402/1,826 (22.02%)	1.06 (0.91–1.23)
**Alzheimer’s disease**	43/4,319 (1.00%)	27,702/1,979,450 (1.40%)	0.61 (0.45–0.83)	41/4,109 (1.00%)	58/4,063 (1.43%)	0.65 (0.44–0.98)
**Vascular dementia**	76/4,301 (1.77%)	34,017/1,990,961 (1.71%)	0.89 (0.71–1.12)	75/4,093 (1.83%)	102/4,052 (2.52%)	0.68 (0.50–0.91)
**Unspecified dementia**	143/4,255 (3.36%)	69,574/1,931,555 (3.60%)	0.80 (0.68–0.94)	134/4,046 (3.31%)	192/3,894 (4.93%)	0.63 (0.50–0.78)
	**Before PSM**			**After PSM**		
**5-year outcomes**	**EXCR group**	**Non-EXCR group**	**HR (95% CI)**	**EXCR group**	**Non-EXCR group**	**HR (95% CI)**
**Composite dementia**	258/4,212 (6.13%)	121,784/1,898,781 (6.41%)	0.80 (0.71–0.91)	240/4,005 (5.99%)	300/3,832 (7.83%)	0.70 (0.59–0.82)
**All-cause mortality**	676/4,348 (15.55%)	349,560/2,013,842 (17.36%)	0.75 (0.70–0.81)	645/4,138 (15.59%)	1,145/4,138 (27.67%)	0.50 (0.45–0.55)
**AMI**	292/2,851 (10.24%)	84,399/1,900,472 (4.44%)	2.00 (1.78–2.24)	279/2,737 (10.19%)	301/3,012 (9.99%)	0.95 (0.81–1.12)
**Stroke recurrence**	169/1,041 (16.23%)	74,830/551,382 (13.57%)	1.10 (0.95–1.28)	160/990 (16.16%)	251/1,243 (20.19%)	0.75 (0.61–0.91)
**Rehospitalisation**	419/1,206 (34.74%)	280,675/1,682,408 (16.68%)	1.91 (1.74–2.10)	411/1,184 (34.71%)	489/1,826 (26.78%)	1.21 (1.06–1.38)
**Alzheimer’s disease**	67/4,319 (1.55%)	38,254/1,979,450 (1.93%)	0.67 (0.53–0.86)	64/4,109 (1.56%)	86/4,063 (2.12%)	0.67 (0.48–0.92)
**Vascular dementia**	110/4,301 (2.56%)	46,082/1,990,961 (2.31%)	0.93 (0.77–1.12)	105/4,093 (2.57%)	125/4,052 (3.08%)	0.76 (0.59–0.98)
**Unspecified dementia**	216/4,255 (5.08%)	97,078/1,931,555 (5.03%)	0.85 (0.74–0.97)	201/4,046 (4.97%)	266/3,894 (6.83%)	0.66 (0.55–0.79)

Abbreviations: AMI, acute myocardial infarction; EXCR, exercise-based cardiac rehabilitation; HR, hazard ratio; PSM, propensity score matching.

**Figure 1:**
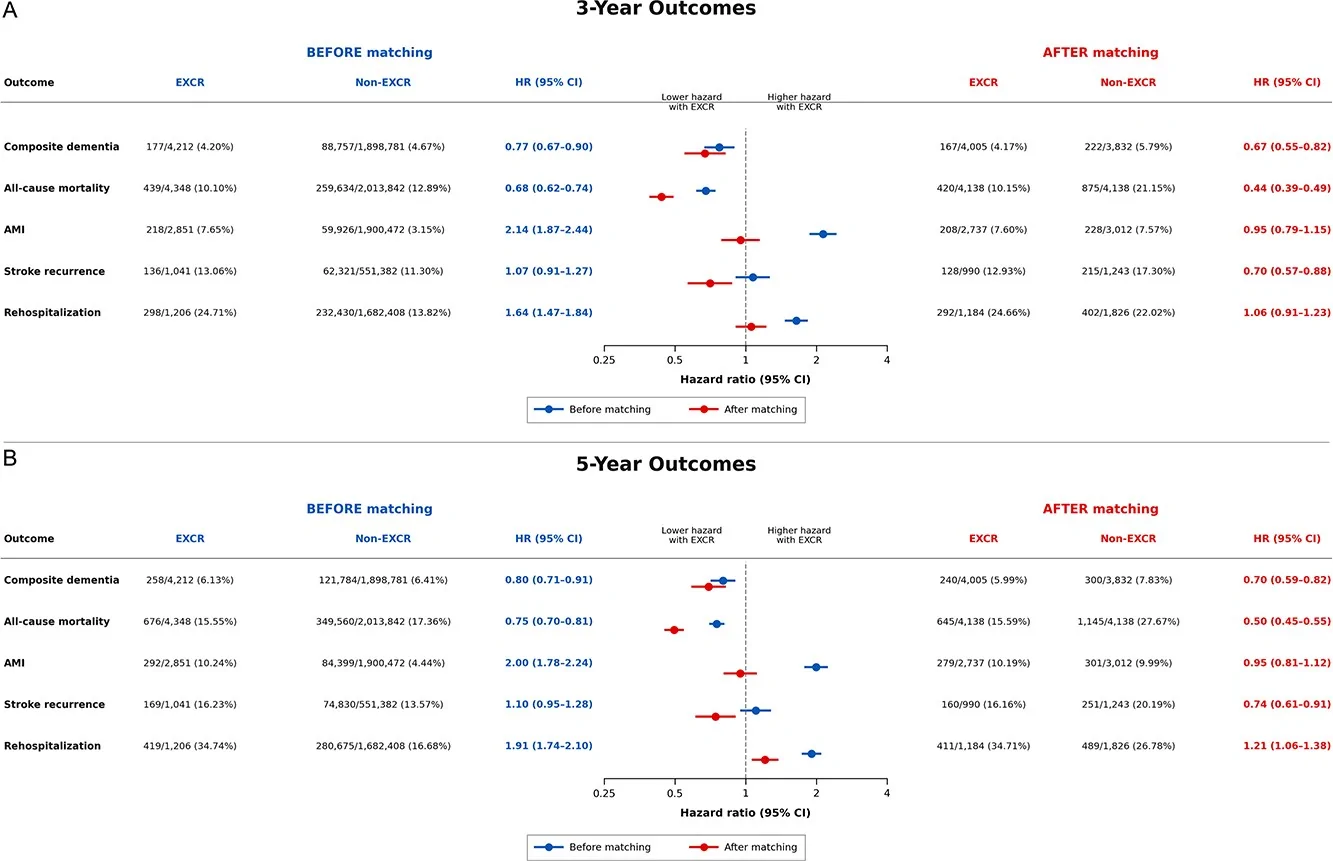
(A) Forest plots of 3-year dementia composite, and clinical outcomes before and after propensity score matching. (B) Forest plots of 5-year dementia composite, and clinical outcomes before and after propensity score matching.

#### Five-year outcomes

Before PSM, EXCR was associated with lower hazards of composite dementia (HR, 0.80; 95% CI, 0.71–0.91), Alzheimer’s disease (HR, 0.67; 95% CI, 0.53–0.86), unspecified dementia (HR, 0.85; 95% CI, 0.74–0.97) and all-cause mortality (HR, 0.75; 95% CI, 0.70–0.81), but with a higher hazard of rehospitalisation (HR, 1.91; 95% CI, 1.74–2.10), compared with no EXCR (Table 2). After PSM, the EXCR group had lower hazards of composite dementia (HR, 0.70; 95% CI, 0.59–0.82), Alzheimer’s disease (HR, 0.67; 95% CI, 0.48–0.92), vascular dementia (HR, 0.76; 95% CI, 0.59–0.98), unspecified dementia (HR, 0.66; 95% CI, 0.55–0.79), all-cause mortality (HR, 0.50; 95% CI, 0.45–0.55) and stroke recurrence (HR, 0.75; 95% CI, 0.61–0.91) compared with the non-EXCR group. Conversely, EXCR was associated with a higher hazard of rehospitalisation (HR, 1.21; 95% CI, 1.06–1.38), while no significant difference was observed for AMI (Table 2; Figure 1).

Formal assessment of the proportional hazards assumption showed no evidence of violation for the 5-year analysis (*P* = .142), whereas a possible departure from proportional hazards was observed at 3 years (*P* = .030). Complementary fixed-time analyses were consistent with the primary findings, with risk ratios for incident composite dementia of 0.72 (95% CI, 0.59–0.88) at 3 years and 0.77 (95% CI, 0.65–0.90) at 5 years (Table S7).

### Sensitivity analyses

#### One-year lagged analysis

After PSM, the EXCR group had lower hazards of composite dementia (HR, 0.79; 95% CI, 0.64–0.97), unspecified dementia (HR, 0.74; 95% CI, 0.59–0.92) and all-cause mortality (HR, 0.61; 95% CI, 0.54–0.69) compared with the non-EXCR group. Conversely, EXCR was associated with a higher hazard of rehospitalisation (HR, 1.55; 95% CI, 1.29–1.85). No significant differences were observed for Alzheimer’s disease, vascular dementia, AMI or stroke recurrence (Table 3; Figure 2; Figure S1).

**Table 3:** Hazard ratios comparing the EXCR group with the non-EXCR group in the 1-year lagged sensitivity analysis with 5-year follow-up, before and after propensity score matching.

Before PSM			
Outcome	EXCR group	Non-EXCR group	HR (95% CI)
**Composite dementia**	188/4,142 (4.54%)	75,237/1,852,234 (4.06%)	0.91 (0.79–1.05)
**All-cause mortality**	483/4,348 (11.11%)	198,952/2,013,842 (9.88%)	0.91 (0.83–1.00)
**AMI**	187/2,746 (6.81%)	54,339/1,870,412 (2.91%)	1.94 (1.68–2.24)
**Stroke recurrence**	88/960 (9.17%)	30,875/507,427 (6.08%)	1.37 (1.11–1.69)
**Rehospitalisation**	249/1,036 (24.03%)	106,154/1,507,887 (7.04%)	2.91 (2.57–3.30)
**Alzheimer’s disease**	49/4,301 (1.14%)	24,033/1,965,229 (1.22%)	0.76 (0.57–1.00)
**Vascular dementia**	77/4,268 (1.80%)	27,765/1,972,644 (1.41%)	1.04 (0.83–1.30)
**Unspecified dementia**	161/4,200 (3.83%)	61,917/1,896,394 (3.26%)	0.96 (0.82–1.12)
**After PSM**			
**Outcome**	**EXCR group**	**Non-EXCR group**	**HR (95% CI)**
**Composite dementia**	172/3,937 (4.37%)	184/3,716 (4.95%)	0.79 (0.64–0.97)
**All-cause mortality**	460/4,138 (11.12%)	657/4,138 (15.88%)	0.61 (0.54–0.69)
**AMI**	178/2,636 (6.75%)	184/2,895 (6.36%)	0.97 (0.79–1.19)
**Stroke recurrence**	83/913 (9.09%)	107/1,099 (9.74%)	0.88 (0.66–1.17)
**Rehospitalisation**	244/1,017 (23.99%)	223/1,560 (14.29%)	1.55 (1.29–1.85)
**Alzheimer’s disease**	46/4,091 (1.12%)	58/4,035 (1.44%)	0.69 (0.47–1.02)
**Vascular dementia**	73/4,061 (1.80%)	76/4,003 (1.90%)	0.84 (0.61–1.16)
**Unspecified dementia**	148/3,993 (3.71%)	170/3,798 (4.48%)	0.74 (0.59–0.92)

Abbreviations: AMI, acute myocardial infarction; EXCR, exercise-based cardiac rehabilitation; HR, hazard ratio; PSM, propensity score matching.

Data are presented as number of events/number of patients with available data (%). Hazard ratios (HRs) were calculated using time-to-event analyses. Denominators may vary across outcomes because of differences in data availability within the network

**Figure 2:**
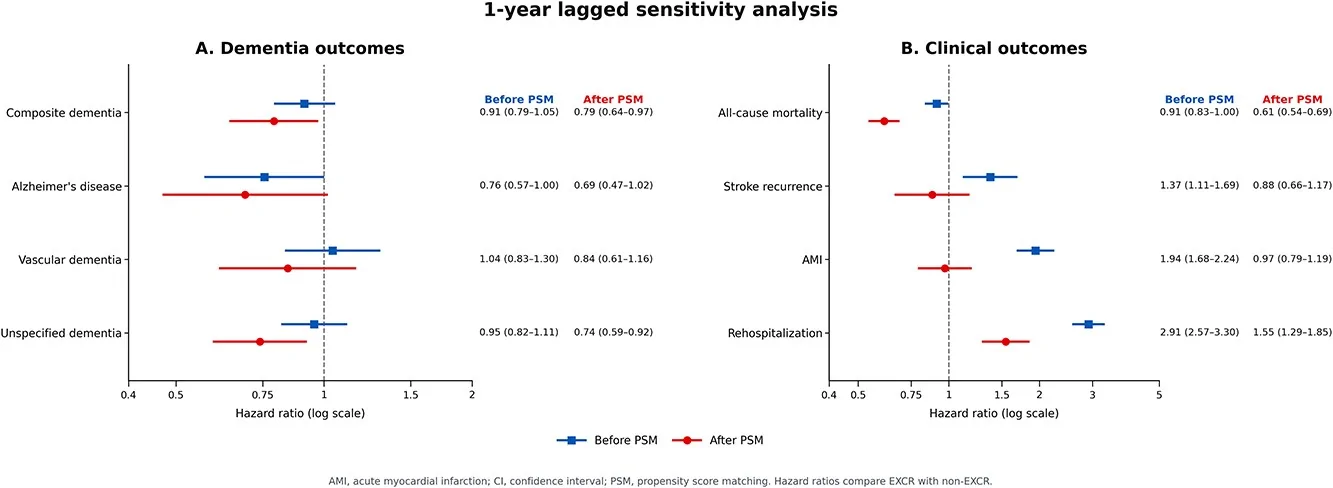
Forest plots of 5-year outcomes from the 1-year lagged sensitivity analysis before and after propensity score matching. Hazard ratios (HRs) and 95% CIs are shown for dementia-related and clinical outcomes. White squares indicate estimates before matching and black squares indicate estimates after matching. The dashed vertical line represents a hazard ratio of 1.0.

#### Thirty-day landmark analysis

After applying a 30-day landmark period, 4161 and 4119 matched patients remained in each cohort for the 3- and 5-year analyses, respectively. Baseline characteristics remained well balanced, with all standardised mean differences below 0.10. The findings remained consistent with the primary analysis. At 3 years, the EXCR group had lower hazards of composite dementia (HR, 0.66; 95% CI, 0.54–0.81) and all-cause mortality (HR, 0.46; 95% CI, 0.41–0.52) compared with the non-EXCR group. Similar associations were observed at 5 years for composite dementia (HR, 0.66; 95% CI, 0.56–0.79) and all-cause mortality (HR, 0.52; 95% CI, 0.47–0.57). Rehospitalisation was more frequent in the EXCR group at both 3 years (HR, 1.28; 95% CI, 1.07–1.54) and 5 years (HR, 1.38; 95% CI, 1.18–1.60) (Table S3).

#### Sensitivity analysis including all stroke-severity categories

In the additional sensitivity analysis including all eligible patients with ischaemic stroke or TIA without exclusion based on NIHSS severity, baseline characteristics were adequately balanced after PSM, with all standardised mean differences < 0.10 (Table S6). The findings remained consistent with the primary analysis. After PSM, EXCR was associated with a lower risk of incident composite dementia at both 3 years (HR, 0.64; 95% CI, 0.53–0.78) and 5 years (HR, 0.67; 95% CI, 0.57–0.79) (Figure S2).

### Subgroup analyses

After PSM, EXCR was associated with a lower risk of incident composite dementia in patients aged ≤ 74 years (HR, 0.61; 95% CI, 0.39–0.94) and ≥ 75 years (HR, 0.69; 95% CI, 0.55–0.87), women (HR, 0.72; 95% CI, 0.52–0.99) and patients with atrial fibrillation (HR, 0.63; 95% CI, 0.42–0.95). Significant associations were also observed among patients without diabetes mellitus (HR, 0.59; 95% CI, 0.43–0.81), with and without chronic ischaemic heart disease (HR, 0.75; 95% CI, 0.56–1.00 and HR, 0.59; 95% CI, 0.36–0.96, respectively), without peripheral artery disease (HR, 0.58; 95% CI, 0.46–0.74) and without chronic kidney disease (HR, 0.60; 95% CI, 0.45–0.79) (Figure 3; Table S4). No statistically significant interactions were observed according to age, sex, diabetes mellitus, atrial fibrillation, chronic ischaemic heart disease, peripheral artery disease or chronic kidney disease (all *P*-interaction > .05) (Table S5). Interaction testing according to hypertension status could not be performed because the outcome estimate for patients without hypertension was unavailable owing to the limited number of events.

**Figure 3:**
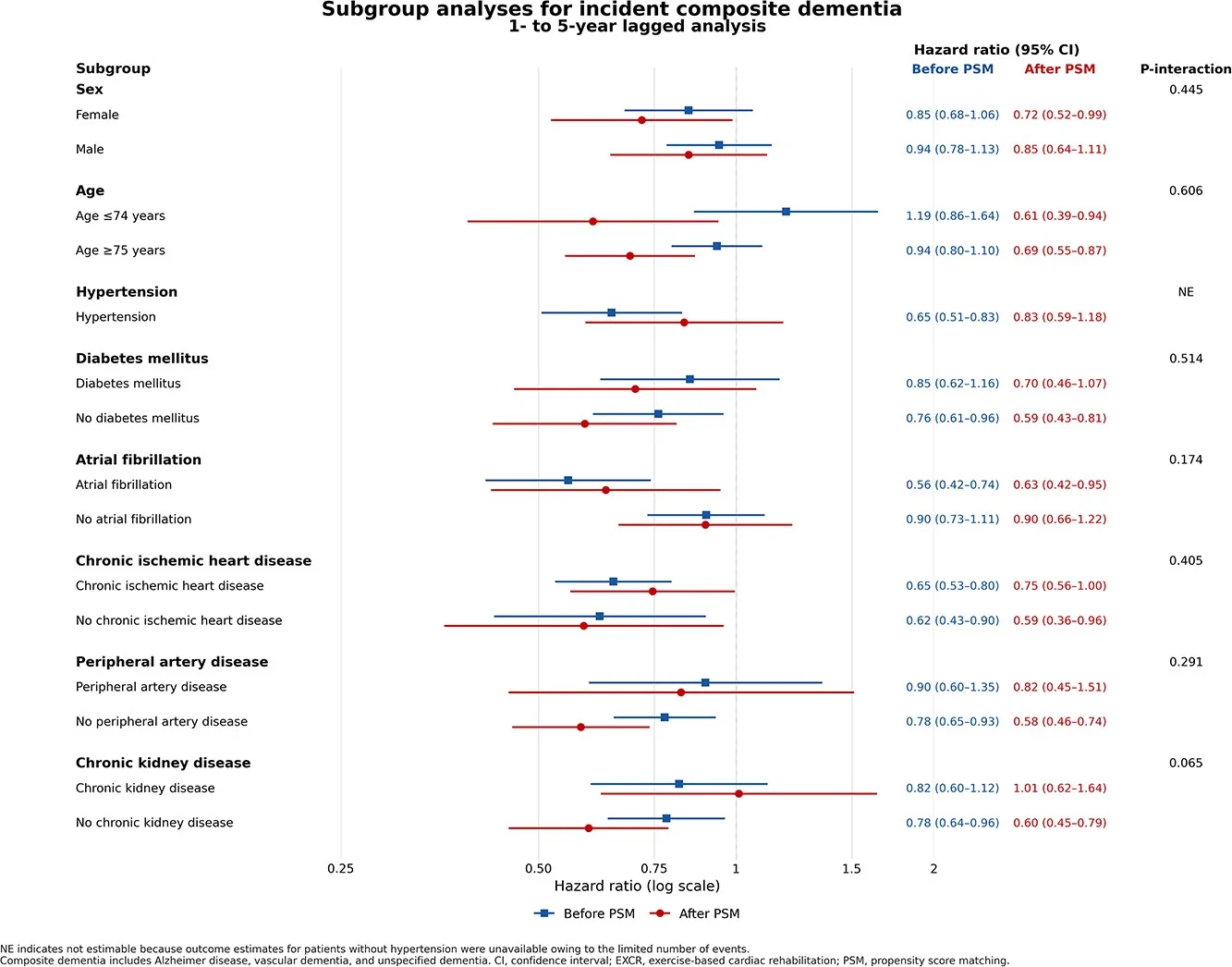
Forest plots of subgroup analyses for the composite dementia outcome in the 1-year lagged sensitivity analysis before and after propensity score matching.

## Discussion

The three main findings of the present study are as follows: (1) in a large propensity score-matched cohort of patients without recorded ICD-10-CM codes indicating NIHSS scores ≥ 16, initiation of EXCR within 6 months after stroke or TIA was associated with lower hazards of incident dementia and all-cause mortality. Associations were also observed for individual dementia subtypes and remained generally consistent across landmark and lag-time sensitivity analyses. Importantly, the association with a lower hazard of composite dementia was confirmed in the additional sensitivity analysis including all eligible patients without applying an NIHSS-based exclusion; (2) subgroup-specific estimates were generally directionally consistent across major demographic and clinical strata, including age, sex, atrial fibrillation, diabetes mellitus, chronic ischaemic heart disease, peripheral artery disease and chronic kidney disease. Although statistical significance was not reached in every subgroup, no significant effect modification was detected; and (3) EXCR was associated with a lower hazard of recurrent stroke, whereas no significant association was observed for acute myocardial infarction. Rehospitalisation was more frequent in the EXCR group at 5 years, an observation that may reflect differences in healthcare contact, clinical surveillance or rehabilitation-related care and therefore warrants cautious interpretation.

In the present study, documented initiation of EXCR was associated with a lower risk of dementia after stroke. These findings are clinically relevant given the persistently high burden of post-stroke cognitive impairment,^17^ which remains common even among patients with relatively limited neurological deficits despite advances in acute stroke treatment and reperfusion therapies.^18^ Cognitive impairment directly attributable to the index stroke should, however, be distinguished from subsequent progressive cognitive decline. The present study assessed newly recorded diagnoses of dementia during follow-up and could not determine whether these diagnoses reflected persistent consequences of the index brain injury, recurrent vascular injury, previously unrecognised cognitive impairment, or an ongoing neurodegenerative or vascular process.^19^ Consistent with our findings, previous studies have linked cardiac rehabilitation, structured exercise interventions and higher levels of physical activity to better cognitive outcomes or a lower risk of dementia.^20^^,^^21^ Furthermore, a recent systematic review including 1085 patients with acute coronary syndrome reported consistent positive effects of exercise-based cardiac rehabilitation on cognitive function.^22^ As reported in the literature, exercise-based cardiac rehabilitation has been shown to improve executive function, working memory and overall cognitive performance, while promoting greater cortical activation and neural efficiency.^20^ Under the assumptions required for causal interpretation, these findings may be compatible with a potential protective effect of EXCR; however, residual confounding, exposure misclassification and selection into rehabilitation remain important alternative explanations. Consistent with our findings, a prospective cohort study of 84,854 participants from the UK Biobank demonstrated an inverse dose–response association between device-measured physical activity and incident dementia, whereby higher levels of moderate-to-vigorous physical activity were associated with a lower risk of dementia.^21^ Similarly, among 223,426 patients with ischaemic stroke, initiating or maintaining regular exercise after stroke was associated with a significantly lower risk of incident dementia compared with those who remained persistently inactive.^23^ Recovery after stroke involves prolonged neuroplastic adaptation and functional brain reorganisation. Exercise-based rehabilitation may support these processes through multiple biological pathways, including improvements in cerebral perfusion, endothelial function, blood–brain barrier integrity and modulation of cardiovascular–brain signalling.^24^ Aerobic exercise has also been associated with increased expression of brain-derived neurotrophic factor (BDNF), a key mediator of synaptic remodelling and neuronal survival and a recent meta-analysis including 687 stroke survivors reported increased circulating BDNF concentrations following exercise interventions, supporting a potential role of exercise in facilitating neuroplastic recovery mechanisms.^25^^,^^26^ Collectively, these observations provide biological context for the association observed in the present study, without establishing that EXCR itself was responsible for the lower incidence of dementia.

In the present study, EXCR was associated with a lower hazard of composite dementia, whereas estimates for individual dementia subtypes varied according to subtype and follow-up window. These findings should be interpreted cautiously given the substantial overlap between dementia subtypes after stroke and the high proportion of unspecified dementia diagnoses in administrative databases.^3^^,^^27–29^ Nevertheless, the association with composite dementia remained directionally consistent in the landmark and 1-year lagged sensitivity analyses. These analyses partially addressed concerns regarding early post-stroke vulnerability, delayed recognition of pre-existing cognitive impairment and reverse causation, although these sources of bias could not be eliminated. This approach is particularly relevant because previous studies have highlighted that cognitive trajectories after stroke are heterogeneous, with distinct mechanisms contributing to early- and delayed-onset post-stroke dementia.^4^^,^^30^^,^^31^ Overall, our findings, taken together with clinical and mechanistic evidence, support a potential role for EXCR in promoting long-term brain health and reducing the risk of incident dementia and adverse clinical outcomes after stroke.

Subgroup-specific estimates were generally directionally consistent with the primary analysis, although statistical significance was not reached in every stratum. Interaction analyses did not identify significant effect modification according to age, sex, atrial fibrillation, diabetes mellitus, chronic ischaemic heart disease, peripheral artery disease or chronic kidney disease; however, the absence of statistically significant interactions should not be interpreted as evidence that the association was identical across subgroups. Atrial fibrillation is a well-established determinant of post-stroke cognitive impairment and dementia, reflecting the combined effects of cardioembolism, cerebral hypoperfusion and shared vascular risk factors.^32^^,^^33^ Peripheral artery disease has been associated with cognitive impairment,^34^^,^^35^ whereas diabetes mellitus,^36^ and ischaemic heart disease^37^ have been linked to cognitive decline and dementia. Collectively, these comorbidities represent markers of diffuse vascular dysfunction^38^ and systemic atherosclerotic and metabolic burden,^39^ which may contribute to cognitive decline through multiple competing pathways. The wider confidence intervals and lack of statistical significance in some strata may reflect limited precision due to smaller subgroup sample sizes rather than genuine differences in the association with EXCR. Nevertheless, subgroup analyses should be considered exploratory and interpreted with caution. Overall, the subgroup findings did not provide evidence of statistically significant effect modification but should not be interpreted as establishing benefit within individual patient categories.

In the present analysis, beyond cognitive outcomes, documented EXCR initiation was also associated with a lower hazard of recurrent ischaemic stroke during follow-up. Recurrent stroke remains a major clinical challenge among stroke survivors and is associated with substantial morbidity and mortality.^40^^,^^41^ Consistent with this, 41% of 28-day survivors experienced a recurrent stroke within 5 years in a large community-based cohort including more than 45,000 individuals with incident stroke.^42^ Importantly, in agreement with our results, analysis of 4899 patients with ischaemic stroke demonstrated that higher rehabilitation frequency during the first year after stroke was associated with lower risks of recurrent stroke and all-cause mortality.^43^ Conversely, EXCR initiation was associated with a higher hazard of rehospitalisation at 5 years. This finding contrasts with a previous study conducted in patients with coronary artery disease, which reported lower readmission rates and a lower adjusted hazard of the composite of all-cause readmission or death among cardiac rehabilitation participants.^44^ Differences in the study population, outcome definitions and patterns of healthcare utilisation may partly account for this discrepancy. Moreover, more frequent contact with healthcare services among EXCR participants may have increased the opportunity for hospital encounters to be captured in EHR data, a recognised source of informed-presence bias.^45^ However, the available data did not permit assessment of the indications for rehospitalisation or confirmation of this hypothesis. Therefore, the association between EXCR and recurrent stroke should be considered hypothesis-generating and interpreted alongside the contrasting rehospitalisation finding.

Taken together, our findings indicate that documented EXCR initiation was associated with lower hazards of dementia, all-cause mortality and recurrent stroke, although this pattern was not observed uniformly across all clinical outcomes. Although these observations require confirmation in prospective studies specifically designed to address residual confounding and treatment selection, they are broadly consistent with evidence supporting structured rehabilitation in recovery and long-term care after cerebrovascular events.^46–48^ However, EXCR should not be interpreted as a substitute for conventional stroke rehabilitation. The available data did not establish whether EXCR was delivered in addition to neurological rehabilitation, nor did they permit comparison of the respective contributions of these rehabilitation approaches. In line with contemporary stroke management guidelines and integrated models of post-stroke care,^49^^,^^50^ these findings provide a rationale for further investigation of EXCR as a possible complementary component of post-stroke care, including its potential relationship with long-term cognitive outcomes.

## Limitations

Several limitations of this study should be acknowledged. First, although the study was structured around an explicit causal contrast, the assumptions required for causal interpretation could not be fully verified using retrospective federated EHR data. These assumptions include consistency, positivity, conditional exchangeability given the measured baseline covariates, accurate classification and temporal ordering of exposure and outcomes, and non-informative censoring. In particular, conditional exchangeability may not have been achieved because relevant determinants of both EXCR initiation and dementia risk including baseline cognitive and functional status, frailty, socioeconomic circumstances, rehabilitation eligibility, healthcare access and adherence were incompletely captured or unavailable. Although heart failure was included among the baseline covariates, New York Heart Association (NYHA) functional class was not available as a sufficiently structured and consistently recorded variable in the federated EHR data; therefore, residual confounding related to heart failure severity cannot be excluded. Propensity score matching could therefore reduce imbalance only in measured baseline characteristics, and the reported hazard ratios should be interpreted as adjusted associations rather than definitive causal effects. In addition, time-to-event estimates may have been affected by informative censoring, inaccuracies in recorded event timing and departures from the proportional hazards assumption, although complementary fixed-time analyses yielded consistent findings.

Second, the specific characteristics of EXCR, including its individual components, session frequency and intensity, duration, timing within the prespecified 6-month exposure window, adherence, completion and rehabilitation setting, were unavailable or could not be standardised across participating healthcare organisations. Consequently, dose–response relationships and the contribution of individual EXCR components could not be evaluated. This is particularly relevant given the multidimensional and individualised nature of post-stroke rehabilitation strategies recommended by contemporary stroke guidelines.^50^ The available data also did not establish whether EXCR was delivered in addition to, or as a substitute for, conventional stroke rehabilitation, or whether patients in the non-EXCR group received other forms of rehabilitation. Moreover, cardiac rehabilitation delivered outside participating healthcare organisations or recorded using different codes may have resulted in exposure misclassification. Third, the TriNetX platform does not provide granular information regarding cognitive assessments, and validated neurocognitive measures such as the Montreal Cognitive Assessment and Mini-Mental State Examination, commonly used for the evaluation of post-stroke cognitive impairment, were not systematically available.^51^ Accordingly, the available data did not allow assessment of specific cognitive domains, such as memory, executive function and attention, or of subtler cognitive decline not resulting in a recorded dementia diagnosis. Furthermore, the absence of a recorded diagnosis before the index cerebrovascular event does not exclude pre-existing but previously unrecognised cognitive impairment or dementia that may have been identified only during subsequent post-stroke care. Although the 1-year lagged analysis reduced the potential for such cases to be misclassified as incident dementia, this possibility could not be completely eliminated. In addition, the available data could not fully distinguish persistent cognitive consequences of the index stroke from subsequent progressive cognitive decline. Similarly, the available ICD-10-CM-based NIHSS categories provided only a coarse approximation of baseline stroke severity and did not capture individual neurological deficits, infarct topography or volume or acute neurological complications.

Finally, despite adequate balance in measured covariates after propensity score matching, residual confounding by indication and selection into EXCR is likely to remain because referral, eligibility and participation in rehabilitation are highly selective processes. Patients initiating EXCR may differ from non-participants in unmeasured or incompletely captured characteristics, including functional independence, frailty, baseline cognitive status, lifestyle, motivation, treatment adherence, socioeconomic resources, social support and access to rehabilitation. The higher cardiovascular comorbidity and medication burden observed in the EXCR group before matching may also reflect greater healthcare engagement and clinical surveillance. Moreover, differences across healthcare organisations in referral practices, rehabilitation availability, follow-up intensity and dementia ascertainment may have introduced referral, access and surveillance biases. Because propensity score matching balances measured characteristics only, these potential sources of confounding preclude definitive causal interpretation of the observed associations.

## Conclusions

Documented initiation of EXCR within 6 months after stroke or transient ischaemic attack was associated with a lower hazard of incident dementia, together with favourable associations for all-cause mortality and recurrent stroke. The association with composite dementia remained generally consistent across multiple sensitivity analyses, including the analysis without an NIHSS-based exclusion, although findings varied across individual dementia subtypes and secondary clinical outcomes. These observations suggest that EXCR may represent a potentially relevant complement to conventional post-stroke rehabilitation; however, they should be interpreted cautiously in view of the observational design and require confirmation in prospective studies specifically designed to address treatment selection, rehabilitation characteristics and residual confounding.

## Supplementary Material

Supplementary_Materials_Post_revision_aakag127

## Data Availability

The data underlying this study were obtained from the TriNetX LIVE research network. The authors do not have permission to share these data; access is subject to TriNetX’s terms and conditions.

## Trial registration

Not applicable because this was an observational study using routinely collected de-identified electronic health record data.
