# Supplementary material for: Exercise-based cardiac rehabilitation after stroke and the risk of incident dementia: an observational study from a global federated network
Source: Eur Stroke J. 2026 Oct 22;11(10):aakag127. doi: 10.1093/esj/aakag127 (PMC13689881; doi:10.1093/esj/aakag127)
Supplement: Supplementary file 1 [file supplementary_materials_post_revision_aakag127.docx]

**SUPPLEMENTARY MATERIALS**

**Exercise-Based Cardiac Rehabilitation After Stroke and the Risk of Incident Dementia: An Observational Study from a Global Federated Network**

Leandra Serio, Amir Askarinejad, Chen Wang, Koohyar Ahmadzadeh, Tianshu Gu,

Riccardo Proietti, Claudio Ferri , Ben Buckley, Gregory Y.H. Lip

| **Supplementary table 1 .** Definitions of Baseline Clinical Covariates and Corresponding Coding Algorithms |  |  |  |
| --- | --- | --- | --- |

| COVARIATE | CODE SYSTEM | CODE(S) | DESCRIPTION |
| --- | --- | --- | --- |
| COMORBIDITY COVARIATES | | | |
| Hypertension | ICD-10-CM | I10–I1A | Hypertensive diseases |
| Diabetes mellitus | ICD-10-CM | E08–E13 | Diabetes mellitus |
| Dyslipidaemia | ICD-10-CM | E78 | Disorders of lipoprotein metabolism and other lipidaemias |
| Atrial fibrillation/flutter | ICD-10-CM | I48 | Atrial fibrillation and flutter |
| Ischemic heart disease | ICD-10-CM | I20–I25 | Ischemic heart diseases |
| Peripheral vascular disease | ICD-10-CM | I73 | Other peripheral vascular diseases |
| Chronic kidney disease | ICD-10-CM | N18 | Chronic kidney disease |
| Heart failure | ICD-10-CM | I50 | Heart failure |
| Neoplasms | ICD-10-CM | C00–D49 | Neoplasms, including malignant, benign, in situ, and uncertain or unspecified behaviour |
| Personal history of nicotine dependence | ICD-10-CM | Z87.891 | Personal history of nicotine dependence |
| Mild cognitive impairment | ICD-10-CM | G31.84 | Mild cognitive impairment of uncertain or unknown etiology |
| STROKE SEVERITY (NIHSS) | | | |
| NIHSS score 0–9 | ICD-10-CM | R29.70 |  |
| NIHSS score 10–15 | ICD-10-CM | R29.710–R29.715 |  |
| MEDICATION COVARIATES | | | |
| Antilipemic agents | Drug class | CV350 | Lipid-lowering agents |
| ACE inhibitors | Drug class | CV800 | ACE inhibitors |
| Beta-blockers | Drug class | CV100 | Beta-blockers |
| Calcium channel blockers | Drug class | CV200 | Calcium channel blockers |
| ARBs | Drug class | CV805 | Angiotensin II inhibitors |
| Diuretics | Drug class | CV700 | Diuretics |
| Other antihypertensives | Drug class | CV490 | Other antihypertensive agents |

**Footnotes:**

- Covariates were defined based on the presence of at least one corresponding code recorded before the index date.
- Stroke severity was approximated using available ICD-10-CM codes corresponding to NIHSS categories. The NIHSS was selected as a validated and widely used measure of neurological deficit severity after stroke. The numerical categories reported in the table reflect the ICD-10-CM severity-code groupings available within the TriNetX platform. Descriptive severity thresholds vary across published classifications; therefore, numerical NIHSS categories are reported without assigning uniform labels such as mild or moderate stroke.
- Patients with recorded ICD-10-CM codes indicating an NIHSS score of 16 or higher were excluded from the primary study population. Patients without a recorded NIHSS code were not excluded on the basis of stroke severity. The NIHSS-based exclusion was removed in the all-stroke-severity sensitivity analysis.
- Medication covariates were defined based on recorded drug exposures before the index date.
- ACE indicates angiotensin-converting enzyme; ARB, angiotensin II receptor blocker; ICD-10-CM, International Classification of Diseases, Tenth Revision, Clinical Modification; NIHSS, National Institutes of Health Stroke Scale.

**Supplementary table 2**. Definitions of Study Variables and Outcomes Codes

| Category | Variable | Code System | Codes | Description |
| --- | --- | --- | --- | --- |
| Study population | Ischemic stroke | ICD-10-CM | I63 | Cerebral infarction |
|  | Transient ischemic attack | ICD-10-CM | G45 | Transient cerebral ischemic attacks and related syndromes |
| Exposure definition | Cardiac rehabilitation | CPT | 93797, 93798, 1013171 | Outpatient cardiac rehabilitation services |
|  | Rehabilitation and nutrition programmes | HCPCS | S9472, S9452 | Rehabilitation programme and nutrition classes |
|  | Exercise counselling | ICD-10-CM | Z71.82 | Exercise counselling |
| Primary outcome | Composite dementia | ICD-10-CM | G30, F01, F03 | Alzheimer’s disease, vascular dementia, or unspecified dementia |
| Secondary outcomes | Mortality | EHR variable | — | All-cause mortality |
|  | Acute myocardial infarction | ICD-10-CM | I21 | Acute myocardial infarction |
|  | Stroke recurrence | ICD-10-CM | I63 | Recurrent cerebral infarction |
|  | Rehospitalization | CPT | 1013659 | Hospital inpatient and observation care services |

**Abbreviations:** CPT, Current Procedural Terminology; EHR, electronic health record; HCPCS, Healthcare Common Procedure Coding System; ICD-10-CM, International Classification of Diseases, Tenth Revision, Clinical Modification; MI, myocardial infarction; NIHSS, National Institutes of Health Stroke Scale.

- Stroke severity was approximated using available ICD-10-CM-based NIHSS codes. The NIHSS was selected because it is a validated and widely used measure of neurological deficit severity after stroke. Patients with recorded codes indicating an NIHSS score of 16 or higher were excluded from the primary analysis. Patients without a recorded NIHSS code were not excluded on the basis of stroke severity.
- Patients in the EXCR group had a recorded exposure to the prespecified EXCR codes between 1 day and 6 months after the index cerebrovascular event. Patients in the non-EXCR group had no recorded exposure to these codes at any time after the index event. The absence of recorded EXCR codes did not necessarily indicate the absence of other forms of neurological or multidisciplinary post-stroke rehabilitation or exclude the possibility of rehabilitation not captured within the participating healthcare organizations.
- Incident outcomes were analyzed after excluding patients with the relevant outcome before the index date or before the beginning of the applicable follow-up window.

**Supplementary Table 3.** Sensitivity analysis using a 30-day blanking / landmark period before and after propensity score matching

|  | BEFORE PSM | | | | AFTER PSM | | | |
| --- | --- | --- | --- | --- | --- | --- | --- | --- |
| Outcome | | **3-Y**  **EXCR group** | **3-Y**  **non-EXCR group** | **HR**  **(95% CI)** | | **3-Y**  **EXCR group** | **3-Y**  **non-EXCR group** | **HR**  **(95% CI)** |
| ****Composite dementia**** | | 168/4,214 (3.99%) | 83,547/2,000,410 (4.18%) | 0.81 (0.70–0.95) | | 159/4,017 (3.96%) | 210/3,793 (5.54%) | 0.66 (0.54–0.81) |
| ****All-cause mortality**** | | 399/4,362 (9.15%) | 218,464/2,139,175 (10.21%) | 0.76 (0.69–0.84) | | 384/4,161 (9.23%) | 749/4,161 (18.00%) | 0.46 (0.41–0.52) |
| ****AMI**** | | 199/2,840 (7.01%) | 54,800/2,009,112 (2.73%) | 2.24 (1.95–2.57) | | 189/2,731 (6.92%) | 221/3,068 (7.20%) | 0.89 (0.74–1.09) |
| ****Stroke recurrence**** | | 125/1,034 (12.09%) | 41,540/567,987 (7.31%) | 1.56 (1.31–1.86) | | 118/984  (11.99%) | 133/1,115 (11.93%) | 0.98 (0.76–1.25) |
| ****Rehospitalization**** | | 215/1,125 (19.11%) | 111,460/1,640,968 (6.79%) | 2.49 (2.18–2.85) | | 211/1,105 (19.10%) | 243/1,704 (14.26%) | 1.28 (1.07–1.54) |
| ****Alzheimer’s disease**** | | 42/4,331  (0.97%) | 25,657/2,098,743 (1.22%) | 0.68 (0.50–0.91) | | 40/4,130  (0.97%) | 59/4,073 (1.45%) | 0.61 (0.41–0.91) |
| ****Vascular dementia**** | | 74/4,311  (1.72%) | 32,425/2,108,937 (1.54%) | 0.95 (0.76–1.20) | | 74/4,113  (1.80%) | 86/4,066 (2.12%) | 0.78 (0.57–1.06) |
| ****Unspecified dementia**** | | 138/4,262 (3.24%) | 66,338/2,039,057 (3.25%) | 0.85 (0.72–1.00) | | 128/4,062 (3.15%) | 177/3,856 (4.59%) | 0.63 (0.50–0.79) |
|  | | **BEFORE PSM** | | | | **AFTER PSM** | | |
| Outcome | | **5-Y**  **EXCR group** | **5-Y**  **non-EXCR group** | **HR**  **(95% CI)** | | **5-Y**  **EXCR group** | **5-Y**  **non-EXCR group** | **HR**  **(95% CI)** |
| Composite dementia | | 249/4,214 (5.91%) | 119,319/2,000,410 (5.96%) | 0.82 (0.73–0.93) | | 232/3,974 (5.84%) | 294/3,769 (7.80%) | 0.66 (0.56–0.79) |
| All-cause mortality | | 636/4,362 (14.58%) | 316,894/2,139,175 (14.81%) | 0.82 (0.76–0.88) | | 609/4,119 (14.79%) | 1,018/4,119 (24.71%) | 0.52 (0.47–0.57) |
| AMI | | 273/2,840 (9.61%) | 81,029/2,009,112 (4.03%) | 2.04 (1.81–2.30) | | 260/2,706 (9.61%) | 261/2,998 (8.71%) | 1.00 (0.84–1.19) |
| Stroke recurrence | | 158/1,034 (15.28%) | 55,013/567,987 (9.69%) | 1.48 (1.27–1.73) | | 146/973  (15.01%) | 172/1,099 (15.65%) | 0.91 (0.73–1.14) |
| Rehospitalization | | 336/1,125 (29.87%) | 163,140/1,640,968 (9.94%) | 2.68 (2.41–2.99) | | 329/1,102 (29.85%) | 333/1,629 (20.44%) | 1.38 (1.18–1.60) |
| Alzheimer’s disease | | 66/4,331  (1.52%) | 36,986/2,098,743 (1.76%) | 0.72 (0.56–0.91) | | 63/4,088  (1.54%) | 84/4,025  (2.09%) | 0.65 (0.47–0.90) |
| Vascular dementia | | 108/4,311 (2.51%) | 45,485/2,108,937 (2.16%) | 0.97 (0.80–1.17) | | 104/4,070 (2.56%) | 148/4,019 (3.68%) | 0.62 (0.48–0.79) |
| Unspecified dementia | | 212/4,262 (4.97%) | 96,212/2,039,057 (4.72%) | 0.88 (0.77–1.00) | | 197/4,020 (4.90%) | 254/3,824 (6.64%) | 0.65 (0.54–0.79) |

***Footnote****: Values are reported as the number of events/total population (%). The 30-day landmark analysis included outcomes occurring from day 30 after the index event through 3 or 5 years of follow-up. Composite dementia included Alzheimer’s disease, vascular dementia, and unspecified dementia. AMI indicates acute myocardial infarction; CI, confidence interval; EXCR, exercise-based cardiac rehabilitation; HR, hazard ratio; PSM, propensity score matching.*

**Supplementary Table** **4**. Composite outcome dementia (Alzheimer; vascular and unspecified dementia) timing 1-5 years in subgroups analyses

| COMPOSITE DEMENTIA | BEFORE PSM | | | | AFTER PSM | | | |
| --- | --- | --- | --- | --- | --- | --- | --- | --- |
|  | **EXCR group**  **Vs**  **Non-EXCR group** | **EXCR group** | **non-EXCR group** | **HR**  **(95% CI)** | **EXCR group**  **Vs**  **Non-EXCR group** | **EXCR group** | **non-EXCR group** | **HR**  **(95% CI)** |
| ****FEMALE**** | 1,625 vs 989,544 | 76 (4.7%) | 45,362 (4.6%) | 0.85 (0.68–1.06) | 1,556 vs 1,444 | 71 (4.6%) | 81 (5.6%) | 0.72 (0.52–0.99) |
| ****MALE**** | 2,528 vs 971,876 | 113 (4.5%) | 36,894 (3.8%) | 0.94 (0.78–1.13) | 2,376 vs 2,246 | 104 (4.4%) | 100 (4.5%) | 0.85 (0.64–1.11) |
| ****≤74 YEARS**** | 2,070 vs 812,253 | 37 (1.8%) | 10,326 (1.3%) | 1.19 (0.86–1.64) | 1,982 vs 1,939 | 34 (1.7%) | 48 (2.5%) | 0.61 (0.39–0.94) |
| ****≥75 YEARS**** | 2,084 vs 1,148,785 | 152 (7.3%) | 71,884 (6.3%) | 0.94 (0.80–1.10) | 1,960 vs 1,771 | 140 (7.1%) | 157 (8.9%) | 0.69 (0.55–0.87) |
| ****DIABETES MELLITUS**** | 776 vs 657,692 | 39 (5.0%) | 36,115 (5.5%) | 0.85 (0.62–1.16) | 751 vs 703 | 39 (5.2%) | 47 (6.7%) | 0.70 (0.46–1.07) |
| ****NO DIABETES MELLITUS**** | 2,172 vs 1,316,250 | 73 (3.4%) | 45,459 (3.5%) | 0.76 (0.61–0.96) | 2,041 vs 1,943 | 63 (3.1%) | 90 (4.6%) | 0.59 (0.43–0.81) |
| ****HYPERTENSION**** | 1,683 vs 1,365,048 | 63 (3.7%) | 71,700 (5.3%) | 0.65 (0.51–0.83) | 1,622 vs 1,532 | 62 (3.8%) | 66 (4.3%) | 0.83 (0.59–1.18) |
| ****NO HYPERTENSION***** | — | — | — | Not estimable | — | — | — | Not estimable |
| ****ATRIAL FIBRILLATION**** | 1,058 vs 418,907 | 46 (4.3%) | 26,155 (6.2%) | 0.56 (0.42–0.74) | 993 vs 915 | 42 (4.2%) | 55 (6.0%) | 0.63 (0.42–0.95) |
| ****NO ATRIAL FIBRILLATION**** | 2,331 vs 1,547,526 | 91 (3.9%) | 53,625 (3.5%) | 0.90 (0.73–1.11) | 2,230 vs 2,113 | 86 (3.9%) | 80 (3.8%) | 0.90 (0.66–1.22) |
| ****CHRONIC ISCHEMIC HEART DISEASE**** | 1,941 vs 531,187 | 93 (4.8%) | 33,354 (6.3%) | 0.65 (0.53–0.80) | 1,844 vs 1,740 | 87 (4.7%) | 99 (5.7%) | 0.75 (0.56–1.00) |
| ****NO CHRONIC ISCHEMIC HEART DISEASE**** | 1,087 vs 1,437,925 | 28 (2.6%) | 47,057 (3.3%) | 0.62 (0.43–0.90) | 1,013 vs 964 | 27 (2.7%) | 39 (4.0%) | 0.59 (0.36–0.96) |
| ****PERIPHERAL ARTERY DISEASE**** | 316 vs 197,404 | 23 (7.3%) | 14,102 (7.1%) | 0.90 (0.60–1.35) | 304 vs 286 | 22 (7.2%) | 20 (7.0%) | 0.82 (0.45–1.51) |
| ****NO PERIPHERAL ARTERY DISEASE**** | 3,241 vs 1,733,208 | 120 (3.7%) | 66,531 (3.8%) | 0.78 (0.65–0.93) | 3,119 vs 2,931 | 113 (3.6%) | 160 (5.5%) | 0.58 (0.46–0.74) |
| ****CHRONIC KIDNEY DISEASE**** | 606 vs 362,370 | 40 (6.6%) | 25,596 (7.1%) | 0.82 (0.60–1.12) | 584 vs 548 | 38 (6.5%) | 29 (5.3%) | 1.01 (0.62–1.64) |
| ****NO CHRONIC KIDNEY DISEASE**** | 2,749 vs 1,604,834 | 91 (3.3%) | 53,967 (3.4%) | 0.78 (0.64–0.96) | 2,590 vs 2,456 | 84 (3.2%) | 117 (4.8%) | 0.60 (0.45–0.79) |

**Limited sample size and insufficient number of events for reliable analysis.*

**Supplementary Table 5.** Interaction analyses for the association between EXCR participation and incident dementia across major clinical subgroups after propensity score matching.

| ****Subgroup comparison**** | ****HR category 1**** | ****HR category 2**** | ****Z statistic**** | ****P for interaction**** |
| --- | --- | --- | --- | --- |
| Female vs male | 0.718 | 0.846 | −0.764 | **0.445** |
| Age ≤74 vs ≥75 years | 0.605 | 0.689 | −0.515 | **0.606** |
| Diabetes vs no diabetes | 0.702 | 0.588 | 0.652 | **0.514** |
| Hypertension vs no hypertension | 0.833 | Not estimable | — | **Not estimable** |
| Atrial fibrillation vs no atrial fibrillation | 0.633 | 0.898 | −1.358 | **0.174** |
| Chronic ischemic heart disease vs no chronic ischemic heart disease | 0.746 | 0.586 | 0.832 | **0.405** |
| Peripheral artery disease vs no peripheral artery disease | 0.824 | 0.580 | 1.056 | **0.291** |
| Chronic kidney disease vs no chronic kidney disease | 1.009 | 0.596 | 1.846 | **0.065** |

***Footnote:*** *P-interaction values were calculated using two-sided Z-tests comparing independent log-transformed hazard ratios derived from the corresponding 95% confidence intervals. Interaction analyses were based exclusively on after-PSM estimates. The P-interaction for hypertension status could not be calculated because the outcome estimates for patients without hypertension were unavailable owing to the limited number of events.*

**Supplementary Table 6**. Baseline characteristics before and after propensity score matching in the sensitivity analysis including all NIHSS severity categories.

|  | BEFORE PSM | | | | AFTER PSM | | | |
| --- | --- | --- | --- | --- | --- | --- | --- | --- |
| Characteristic | **EXCR group (N=4,362)** | **Non-EXCR group (N=1,911,912)** | **P value** | **Std diff** | **EXCR group (N=4,271)** | **Non-EXCR group (N=4,271)** | **P value** | **Std diff** |
| DEMOGRAPHICS |  |  |  |  |  |  |  |  |
| Age at index, years | 64.8 ± 13.6 | 66.3 ± 15.5 | <0.001 | 0.107 | 64.9 ± 13.6 | 65.0 ± 14.3 | 0.684 | 0.009 |
| White | 3,451 (79.4%) | 1,061,848 (59.1%) | <0.001 | 0.453 | 3,389 (79.3%) | 3,421 (80.1%) | 0.389 | 0.019 |
| Male | 2,635 (60.7%) | 867,192 (48.2%) | <0.001 | 0.251 | 2,577 (60.3%) | 2,551 (59.7%) | 0.566 | 0.012 |
| DIAGNOSES |  |  |  |  |  |  |  |  |
| Hypertensive diseases | 3,816 (87.8%) | 682,900 (38.0%) | <0.001 | 1.205 | 3,746 (87.7%) | 3,812 (89.3%) | 0.025 | 0.048 |
| Diabetes mellitus | 1,836 (42.3%) | 320,180 (17.8%) | <0.001 | 0.554 | 1,807 (42.3%) | 1,815 (42.5%) | 0.861 | 0.004 |
| Dyslipidemia | 3,383 (77.9%) | 493,220 (27.4%) | <0.001 | 1.171 | 3,318 (77.7%) | 3,347 (78.4%) | 0.449 | 0.016 |
| Atrial fibrillation and flutter | 1,584 (36.5%) | 161,076 (9.0%) | <0.001 | 0.695 | 1,551 (36.3%) | 1,545 (36.2%) | 0.893 | 0.003 |
| Ischemic heart diseases | 3,086 (71.0%) | 294,662 (16.4%) | <0.001 | 1.320 | 3,031 (71.0%) | 3,144 (73.6%) | 0.006 | 0.059 |
| Other peripheral vascular diseases | 574 (13.2%) | 83,570 (4.6%) | <0.001 | 0.304 | 569 (13.3%) | 563 (13.2%) | 0.848 | 0.004 |
| Chronic kidney disease | 1,070 (24.6%) | 156,720 (8.7%) | <0.001 | 0.437 | 1,059 (24.8%) | 1,085 (25.4%) | 0.516 | 0.014 |
| Heart failure | 1,778 (40.9%) | 149,892 (8.3%) | <0.001 | 0.817 | 1,741 (40.8%) | 1,784 (41.8%) | 0.345 | 0.020 |
| Neoplasms | 1,401 (32.3%) | 348,151 (19.4%) | <0.001 | 0.298 | 1,387 (32.5%) | 1,384 (32.4%) | 0.945 | 0.002 |
| Personal history of nicotine dependence | 1,547 (35.6%) | 118,595 (6.6%) | <0.001 | 0.761 | 1,508 (35.3%) | 1,459 (34.2%) | 0.265 | 0.024 |
| Mild cognitive impairment | 114 (2.6%) | 14,877 (0.8%) | <0.001 | 0.138 | 111 (2.6%) | 103 (2.4%) | 0.580 | 0.012 |
| NIHSS SCORE |  |  |  |  |  |  |  |  |
| NIHSS score 0–9 | 793 (18.3%) | 2,233 (0.1%) | <0.001 | 0.661 | 728 (17.0%) | 692 (16.2%) | 0.295 | 0.023 |
| NIHSS score 10–19 | 174 (4.0%) | 637 (<0.1%) | <0.001 | 0.285 | 148 (3.5%) | 157 (3.7%) | 0.600 | 0.011 |
| NIHSS score 20–29 | 68 (1.6%) | 301 (<0.1%) | <0.001 | 0.175 | 60 (1.4%) | 71 (1.7%) | 0.333 | 0.021 |
| NIHSS score 30–39 | 10 (0.2%) | 64 (<0.1%) | <0.001 | 0.066 | 10 (0.2%) | 10 (0.2%) | 1.000 | <0.001 |
| NIHSS score 40–42 | 10 (0.2%) | 10 (<0.1%) | <0.001 | 0.068 | 10 (0.2%) | 0 (0.0%) | 0.002 | 0.069 |
| MEDICATIONS |  |  |  |  |  |  |  |  |
| Antilipemic agents | 3,704 (85.3%) | 427,336 (23.8%) | <0.001 | 1.570 | 3,632 (85.0%) | 3,601 (84.3%) | 0.352 | 0.020 |
| ACE inhibitors | 2,040 (47.0%) | 283,043 (15.7%) | <0.001 | 0.715 | 2,011 (47.1%) | 2,064 (48.3%) | 0.251 | 0.025 |
| Beta-blockers/related | 3,524 (81.1%) | 426,433 (23.7%) | <0.001 | 1.405 | 3,453 (80.8%) | 3,483 (81.5%) | 0.406 | 0.018 |
| Calcium channel blockers | 2,573 (59.2%) | 299,911 (16.7%) | <0.001 | 0.976 | 2,512 (58.8%) | 2,501 (58.6%) | 0.809 | 0.005 |
| Angiotensin II inhibitors | 1,092 (25.1%) | 164,430 (9.1%) | <0.001 | 0.434 | 1,073 (25.1%) | 1,086 (25.4%) | 0.746 | 0.007 |
| Diuretics | 2,871 (66.1%) | 357,771 (19.9%) | <0.001 | 1.055 | 2,821 (66.1%) | 2,905 (68.0%) | 0.053 | 0.042 |
| Other antihypertensives | 1,737 (40.0%) | 198,092 (11.0%) | <0.001 | 0.705 | 1,709 (40.0%) | 1,776 (41.6%) | 0.140 | 0.032 |
| Platelet aggregation inhibitors | 3,814 (87.8%) | 403,784 (22.5%) | <0.001 | 1.742 | 3,742 (87.6%) | 3,695 (86.5%) | 0.130 | 0.033 |
| Anticoagulants | 3,820 (87.9%) | 366,708 (20.4%) | <0.001 | 1.844 | 3,747 (87.7%) | 3,780 (88.5%) | 0.270 | 0.024 |

***Footnote:*** *Data are presented as mean ± standard deviation or n (%). The sensitivity analysis included patients across all available NIHSS severity categories. Propensity score matching incorporated the grouped NIHSS categories shown in the table, together with demographic characteristics, comorbidities, and medication use. Covariate balance after matching was assessed using absolute standardized differences, with values <0.10 indicating adequate balance. PSM indicates propensity score matching; EXCR, exercise-based cardiac rehabilitation; NIHSS, National Institutes of Health Stroke Scale; ACE, angiotensin-converting enzyme; Std diff, standardized difference.*

**Supplementary Table 7**. Complementary fixed-time analyses and assessment of the proportional hazards assumption for incident composite dementia after propensity score matching

| Time point | EXCR, n/N (%) | Non-EXCR, n/N (%) | Risk ratio (95% CI) | Odds ratio (95% CI) | Proportional hazards test, P value |
| --- | --- | --- | --- | --- | --- |
| 3 years | 167/4,005 (4.17) | 222/3,832 (5.79) | 0.72 (0.59–0.88) | 0.71 (0.58–0.87) | .030 |
| 5 years | 240/4,005 (5.99) | 300/3,832 (7.83) | 0.77 (0.65–0.90) | 0.75 (0.63–0.90) | .142 |

**Footnote:** *Fixed-time risks were calculated as the proportion of patients with incident composite dementia during the specified follow-up window among patients without the outcome before the beginning of the time window. Risk ratios and odds ratios compare EXCR with non-EXCR. The proportional hazards assumption was assessed for the Cox model of incident composite dementia. CI, confidence interval; EXCR, exercise-based cardiac rehabilitation*

**Supplementary Figure 1.** Cumulative incidence of composite dementia and dementia subtypes in the 1- to 5-year lagged analysis.
Bars represent the percentage of patients developing each outcome in the propensity score–matched cohorts. EXCR indicates exercise-based cardiac rehabilitation.

**Supplementary Figure 2 :** Sensitivity analysis of composite dementia at 3 and 5 years across all available NIHSS severity categories, before and after propensity score matching.

**Alternative Text:**

**Supplementary Figure 1.**

Alternative text: Bar chart comparing the cumulative incidence of composite dementia and dementia subtypes between EXCR and non-EXCR patients in the propensity score-matched 1- to 5-year lagged analysis. Composite and unspecified dementia were less frequent in the EXCR group, while the proportions of Alzheimer’s disease and vascular dementia were similar between groups.

**Supplementary Figure 2.**

Alternative text: Forest plot comparing the hazard of composite dementia between EXCR and non-EXCR patients at 3 and 5 years in the sensitivity analysis including all available NIHSS severity categories, before and after propensity score matching. EXCR was associated with a lower hazard of composite dementia at both follow-up periods, with stronger associations after matching
